# Late and very late relapsed acute lymphoblastic leukemia: clinical and molecular features, and treatment outcomes

**DOI:** 10.1038/s41408-021-00516-1

**Published:** 2021-07-02

**Authors:** Ibrahim Aldoss, Raju Pillai, Dongyun Yang, Lixin Yang, Shukaib Arslan, Sally Mokhtari, Monzr M. Al Malki, Amandeep Salhotra, Shilpa Shahani, Haris Ali, Matthew Mei, Andrew Artz, David Snyder, Michelle Afkhami, Saro Armenian, Anthony Stein, Guido Marcucci, Stephen J. Forman, Ryotaro Nakamura, Vinod Pullarkat

**Affiliations:** 1 Department of Hematology and Hematopoietic Cell Transplantation, Gehr Family Center for Leukemia Research, City of Hope, Duarte, CA USA; 2 Department of Pathology, City of Hope, Duarte, CA USA; 3 Department of Computational and Quantitative Medicine, City of Hope, Duarte, CA USA; 4 Department of Pediatrics, City of Hope, Duarte, CA USA

**Keywords:** Medical research, Cancer genomics

Acute lymphoblastic leukemia (ALL) often relapse early after the treatment course, within the first two years from the initial diagnosis [1–3]. Cases of late relapse (LR) occurring in ≥5 years from initial diagnosis have been reported infrequently [4–6]. However, there is no exact definition for late/very late relapse of ALL. Vora et al., reported 12 cases (~1%) with very-late relapse (VLR), defined as >10 years from the time of complete remission (CR), among 1134 children treated on the Medical Research Council (MRC) ALL trials [5]. The MRC UKALLXII/ECOG E2993 reported that 2.8% (*n* = 21) of all relapses in adult ALL patients were late (≥5 years from remission) [6]. Rizzari et al. showed that LR ALL (≥5 years) occurred in 2.9% of Italian children, with a median onset of 6.1 years (range: 5.8–13.7) [4]. It remains unknown if LR results from recurrence of the original leukemia or from a second de novo ALL that is clonally distinct from to the original malignancy. To better understand the clonal origin of LR-ALL and examine treatment outcomes, we analyzed genetic features and clinical outcomes including response to novel agents (i.e., blinatumomab and inotuzumab) [7, 8], and/or allogeneic hematopoietic cell transplantation (alloHCT) in patients treated for ALL at City of Hope (COH).

Of 1280 patients with ALL treated at COH from 2000 to 2020, we identified 36 patients who had their first relapse in ≥5 years after their original diagnosis. This study was approved by our Institutional Review Board. We defined LR and VLR as relapse occurring 5–9 years and ≥10 years after the initial ALL diagnosis, respectively.

Available archived paired DNA samples (collected at diagnosis and relapse, *n* = 3) were analyzed by next generation sequencing (NGS) for genomic alterations and clonal relationship. Supplementary Table 1 shows the clinically significant pathogenic variants and variants of unknown significance from three pairs of samples. Supplementary Fig. 1 is the schematic presentation of clinically significant variants and variants of unknown significance on a genomic scale. Genomic alterations detected were compared between the time points for each patient. Descriptive statistics were used for patient and leukemia characteristics by LR-ALL and VLR-ALL. *χ*^2^ and Wilcoxon tests were used to examine differences in the categorical and continuous characteristics by two groups of patients, respectively. All tests were two-sided at a 0.05 level. Analysis was conducted using SAS version 9.4 (SAS Institute, Cary, NC).

Patient and leukemia characteristics (*n* = 36) are shown in Table 1. Briefly, among patients with LR-ALL, the median latency period from the original diagnosis to the first relapse was 7 years (range: 5–28). There were 12 patients (33%) who had their first relapse ≥ 10 years after the initial diagnosis, with a median of 20 years (range: 10–28), and 24 patients who relapsed in 5–9 years of post-original diagnosis, with a median of 6 years (range: 5–9). With the exception of one patient, none of the LR-ALL or VLR-ALL cases had their first relapse occurred after alloHCT.

**Table 1:** Patient and leukemia characteristics.

	Late relapse (5–9 years) (*N* = 24)	Very late relapse (≥10 years) (*N* = 12)	Total (*N* = 36)	*P* value
Age at initial diagnosis				0.83
Median (Range)	16 (4–35)	16 (3–40)	16 (3–40)	
Interquartile range	11, 23	10, 23	11, 23	
Age at first relapse				0.005
Median (Range)	23 (9–42)	35 (22–51)	25 (9–51)	
Interquartile range	17, 29	26, 41	19, 35	
Sex				1.00
Female	7 (29.2%)	4 (33.3%)	11 (30.6%)	
Male	17 (70.8%)	8 (66.7%)	25 (69.4%)	
Race				0.54
Asian	1 (4.2%)	0 (0%)	1 (2.8%)	
Hispanic	11 (45.8%)	9 (75%)	20 (55.6%)	
Unknown	1 (4.2%)	0 (0%)	1 (2.8%)	
White	10 (41.7%)	3 (25%)	13 (36.1%)	
Black	1 (4.2%)	0 (0%)	1 (2.8%)	
Phenotype at diagnosis				1.00
B cell	21 (87.5%)	11 (91.7%)	32 (88.9%)	
T cell	3 (12.5%)	1 (8.3%)	4 (11.1%)	
Times from first diagnosis to 1st relapse (years)				
Median (Range)	6 (5–9)	20 (10–28)	7 (5–28)	
Interquartile range	5, 7	13, 23	6, 13	
CNS				0.47
NO	19 (79.2%)	9 (75%)	28 (77.8%)	
YES (diagnosis + relapse)	0 (0%)	1 (8.3%)	1 (2.8%)	
YES (relapse)	5 (20.8%)	2 (16.7%)	7 (19.4%)	
WBC count at diagnosis (1000/µL)				0.96
Median (Range)	5 (1–178)	6 (1–55)	5 (1–178)	
Interquartile range	3, 26	3, 30	3, 30	
WBC at first relapse				0.36
Median (Range)	7 (1–158)	30 (2–219)	9 (1–219)	
Interquartile range	4, 17	7, 108	6, 102	
Cytogenetics at diagnosis				0.62
*KMT2A (MLL)*	1 (4.3%)	1 (8.3%)	2 (5.6%)	
Normal Karyotype	8 (34.8%)	2 (16.7%)	10 (27.8%)	
Others	4 (17.4%)	1 (8.3%)	5 (13.9%)	
Ph+	1 (4.3%)	1 (8.3%)	2 (5.6%)	
Unknown/Missing	10 (41.7%)	7 (58.3%)	17 (47.2%)	
Cytogenetics at 1st relapse				0.92
Complex	2 (8.3%)	1 (8.3%)	3 (8.3%)	
*KMT2A (MLL)*	2 (8.3%)	1 (8.3%)	3 (8.3%)	
NK	10 (41.7%)	4 (33.3%)	14 (38.9%)	
Others	4 (16.7%)	1 (8.3%)	5 (13.9%)	
Ph+	1 (4.2%)	2 (16.7%)	3 (8.3%)	
Unknown/Missing	5 (20.8%)	3 (25%)	8 (22.2%)	
Location of 1st relapse				
BM	20 (83.3%)	8 (66.7%)	28 (77.8%)	0.21
Combined	3 (12.5%)	1 (8.3%)	4 (11.1%)	
EMD	1 (4.2%)	3 (25%)	4 (11.1%)	

Leukemia lineage at the time of initial diagnosis was B-cell in most patients (*n* = 32, 89%), and the remaining cases were T-cell lineage ALL (*n* = 4). No instances of lineage switch were detected at relapse. Among the 18 cases with available cytogenetics data at initial diagnosis and first relapse, seven patients had different cytogenetics at first relapse. There were three cases with *KMT2A* gene rearrangement *(KMT2Ar)* detected at relapse. Among these, one patient had *KMT2Ar* detected at initial diagnosis, one patient did not have *KMT2Ar* at initial diagnosis, and one patient had unknown initial cytogenetics diagnosis (Table 1).

Refer to Supplementary Table 2 for initial and first salvage therapies in LR-ALL and administered novel therapies. A flow chart explaining the sequence of treatments is shown in Supplementary Fig. 2. The CR2 rate with salvage therapy at the time of first relapse was 93% (*n* = 33). Three patients required 2 (*n* = 2) or 3 (*n* = 1) cycles of re-induction to attain CR2. Novel therapies administered upon late relapse were blinatumomab (*n* = 9; CR = 7), inotuzumab (*n* = 2; CR = 2) and chimeric antigen receptor (CAR) T cells (*n* = 3; CR = 1). Post-relapse, 28 (78%) patients underwent alloHCT, of whom 20 (71%) patients were transplanted in CR2, 7 (25%) were transplanted in CR3 and one was transplanted after the second relapse.

With a median follow-up from first relapse of 6 years (range: 0.2–20.7), median leukemia-free survival (LFS) and overall survival (OS) were 4.4 years (range: 1.9–6.3) and 14.9 years (range: 5.5–NE) for all patients, respectively. No difference in 5-year LFS (43% vs. 56%, *p* = 0.83) and OS (73% vs. 63%, *p* = 0.70) were observed between patients with LR-ALL and VLR-ALL. (Fig. 1).

**Fig. 1:**
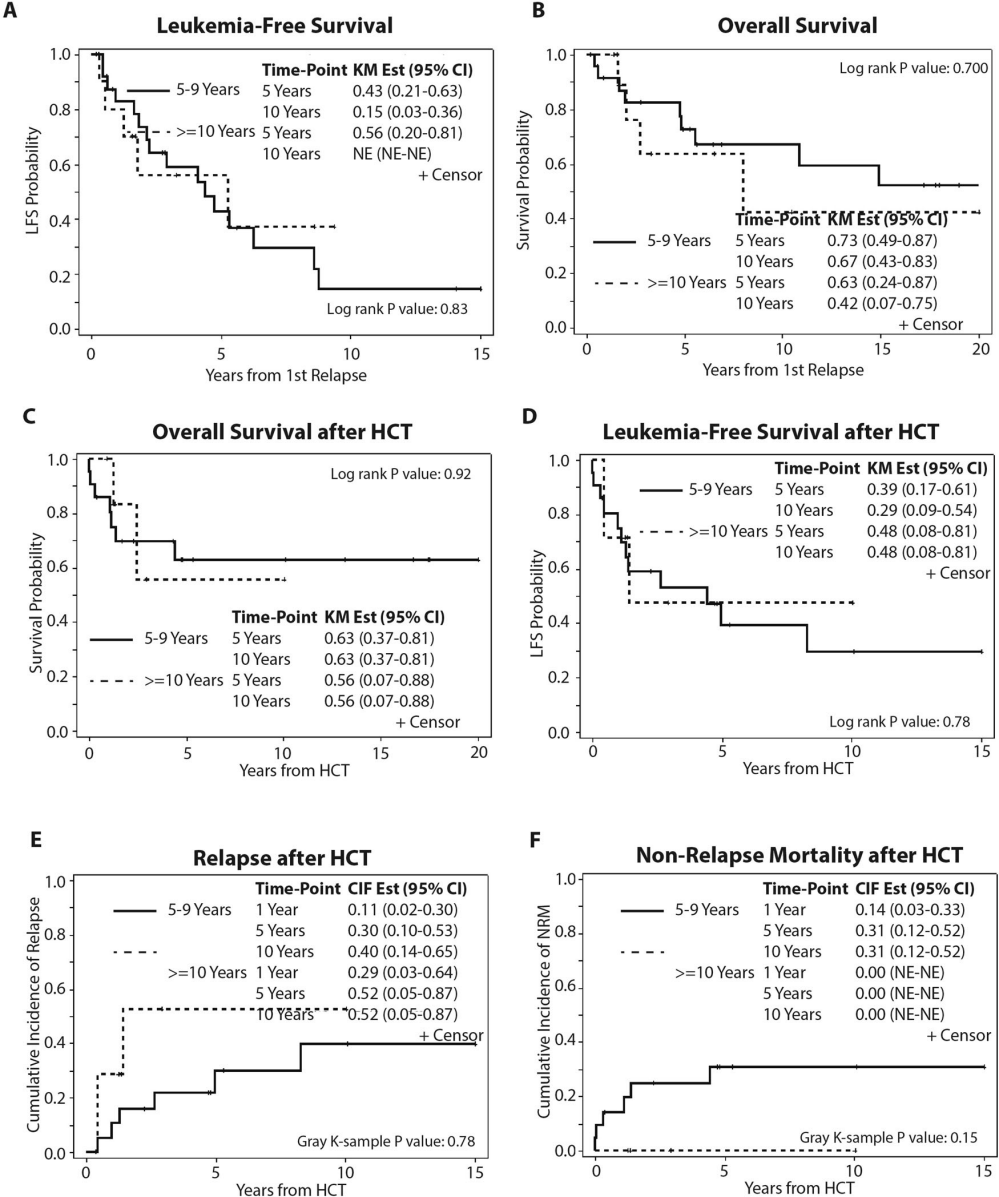
Outcomes of late and very late relapse outcomes. **A** Leukemia-free survival after late relapse. **B** Overall survival after late relapse. **C** Overall survival after HCT. **D** Leukemia-free survival after HCT, **E** Relapse after HCT, and **D** Non-relapse mortality after HCT. Overall survival and leukemia-free survival were compared using Kaplan–Meier curves and log-rank tests. Non-relapse mortality and time to relapse were assessed using cumulative incidence curves.

The median follow-up for patients who underwent alloHCT post-first relapse was 4.7 years (range: 0.4–20.7) from the date of transplant. Non-relapse mortality (NRM) at 1 and 5 years were 11% and 24%, respectively. Relapse rate post-HCT were 16% and 35% at 1 and 5 years, respectively. LFS and OS at 5 years were 40% and 61%, respectively. No difference was observed in OS (*p* = 0.92), LFS (*p* = 0.78), relapse rate (*p* = 0.29), or NRM (*p* = 0.15) between patients with LR-ALL and VLR-ALL following alloHCT (Fig. 1). Land-mark analysis at 1-year of post 1st relapse, no difference in OS between patients who did or did not undergo alloHCT (Supplementary Fig. 3).

Three paired samples obtained at diagnosis and relapse were tested by an NGS panel. The latency periods from diagnosis until relapse were 5, 5, and 10 years. In all three cases, the mutation profiles were different and did not support a clonal relationship, thereby indicating distinct genetics between the original and LR-ALL (Supplementary Table 2 and Supplementary Fig. 3).

Herein, we have described a cohort of patients with LR-ALL (*n* = 36), a third of whom had their first relapse in ≥10 years after their original diagnosis. Interestingly, in two cases, ALL relapse occurred in ~30 years after the initial diagnosis, raising the question “if relapse was of the original disease or from a de novo ALL?”. In our cohort, LR-ALL occurred mostly in patients who did not undergo alloHCT consolidation, suggesting that LR-ALL might be more relevant to chemotherapy-based treatments.

In our cohort, we did not observe any characteristic differences between LR-ALL and VRL-ALL and no unique patterns of leukemia genetics were detected. We suspect that a large subset of LR- ALL patients in our cohort had a true relapse rather than a de novo ALL, supported by similar lineage (B-cell or T-cell) and cytogenetics profile (over half of the cases) at the time of first relapse.

Interestingly, in some cases, *KMT2Ar* and complex cytogenetics were detected as new findings compared to the initial cytogenetics; increasing the possibility of de novo secondary disease from prior exposure to chemotherapy (i.e., therapy-related ALL) [9]. Our hypothesis is further supported by our NGS analysis of paired samples (*n* = 3) showing non-overlapping pathogenic mutations between the diagnosis and relapse samples. However, this finding could also be related to the known phenomena of genetic evolution of leukemia at the time of relapse [10], or the possibility of previously undetectable small clones contributing to late relapse.

In a study by Vora et al., molecular analysis of 8 cases with VLR-ALL showed identical TCR or IgH gene rearrangement at diagnosis and relapse, supporting the concept that LR-ALL could derive from the original clones [5]. In contrast, Ford et al., reported LR-ALL in two cases with *TEL-AML1* ALL that was not derived from the initial clone; and postulated that relapse was possibly independent/de novo and emerged form dormant pre-leukemic clones [11].

In our cohort, LR-ALL remained sensitive to conventional chemotherapy as CR rate was 93% in response to first salvage regimen, and the majority of patients were able to proceed to alloHCT. A subset of our patients received various novel therapies and attained remission. Overall long-term outcomes were encouraging, with over half the patients surviving beyond 5 years from their LR-ALL.

Our study has limitations related to its small size, retrospective nature, and availability of only a handful of paired samples suitable for genetic analysis that could provide additional insight into the origin of LR-ALL. However, our patients with LR-ALL and VLR-ALL had high response rates to re-induction therapy and encouraging outcomes were observed with novel therapies and alloHCT. Larger analysis of paired samples is warranted to gain more insight into the origin of this entity.

## Supplementary information

Supplementary Figure 1

Supplementary Figure 2

Supplementary Figure 3

Supplemental Tables
