# Supplementary figures and images for: Late and very late relapsed acute lymphoblastic leukemia: clinical and molecular features, and treatment outcomes
Source: Blood Cancer J. 2021 Jul 2;11(7):125. doi: 10.1038/s41408-021-00516-1 (PMC8253853; doi:10.1038/s41408-021-00516-1)

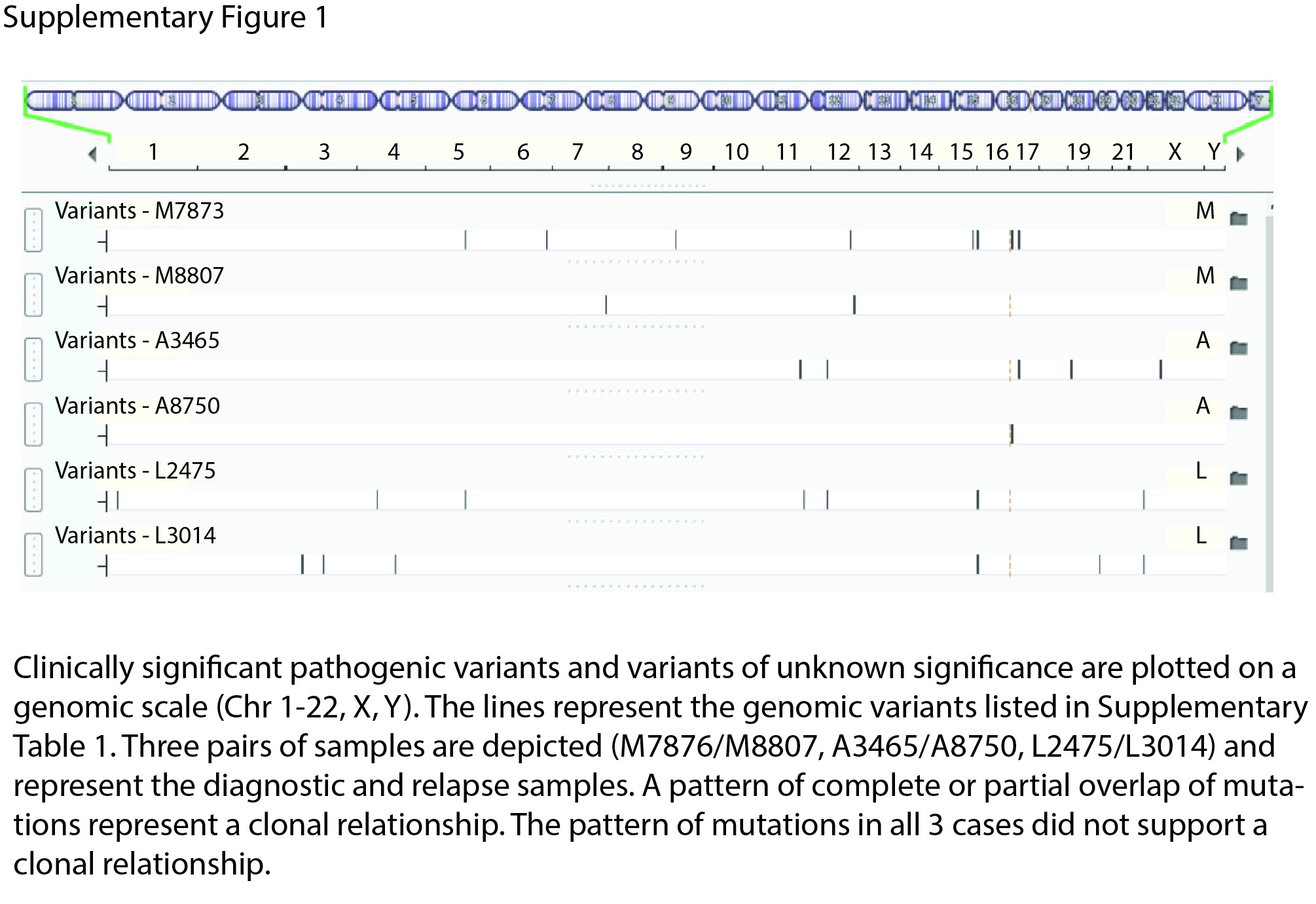

Supplement: Supplementary file 1 — Supplementary Figure 1 [file 41408_2021_516_MOESM1_ESM.jpg]

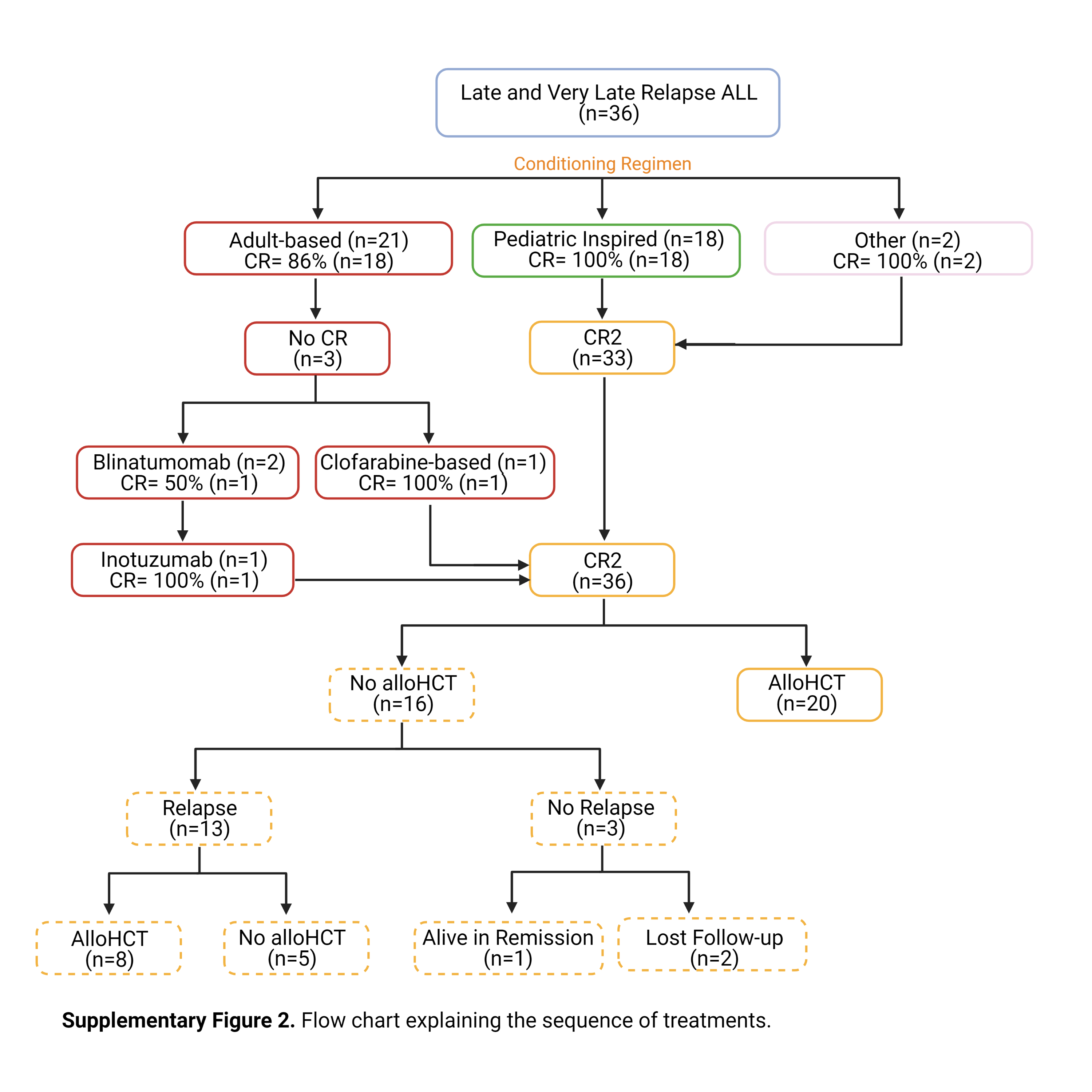

Supplement: Supplementary file 2 — Supplementary Figure 2 [file 41408_2021_516_MOESM2_ESM.jpg]

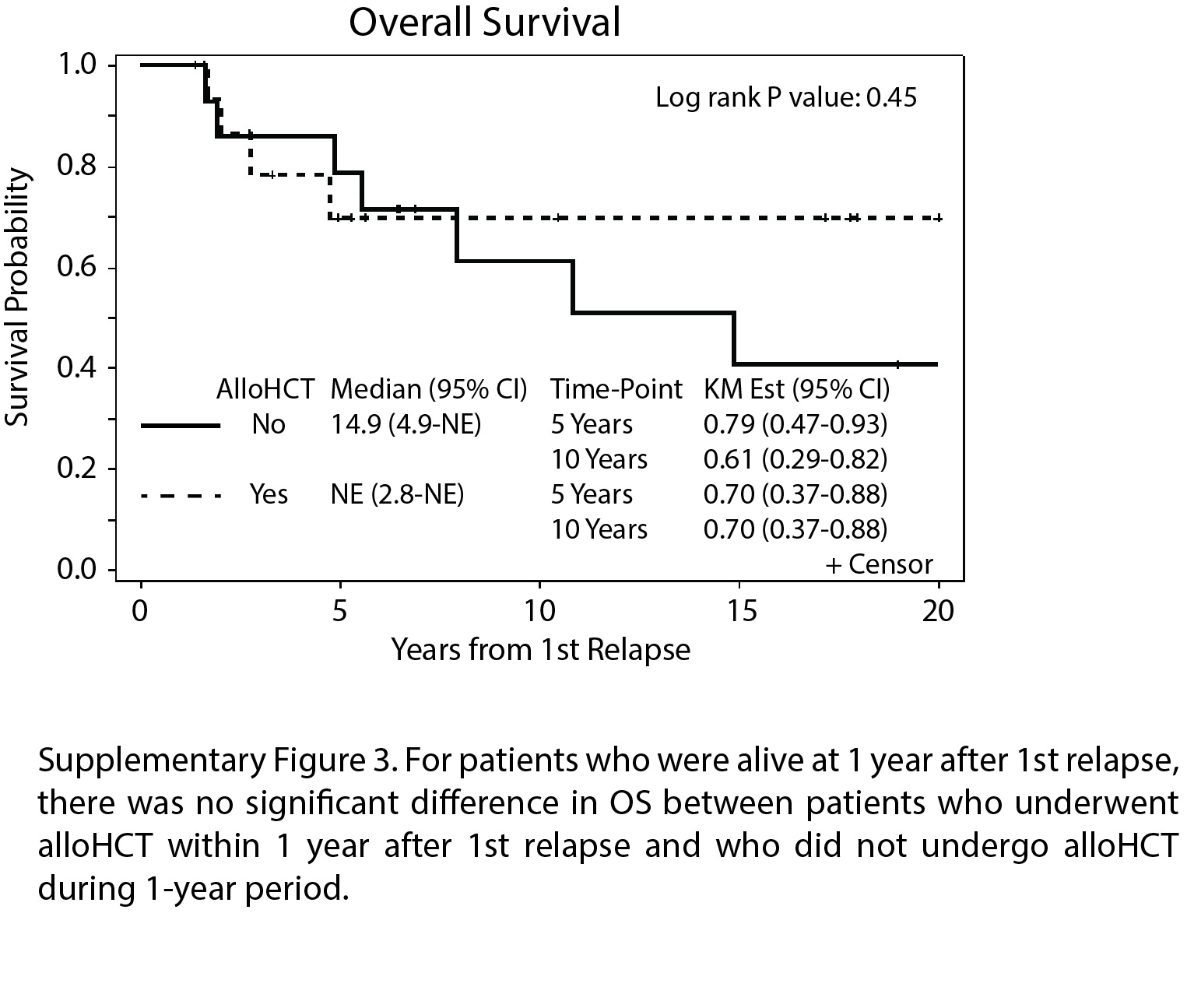

Supplement: Supplementary file 3 — Supplementary Figure 3 [file 41408_2021_516_MOESM3_ESM.jpg]
