# Supplementary material for: Late and very late relapsed acute lymphoblastic leukemia: clinical and molecular features, and treatment outcomes
Source: Blood Cancer J. 2021 Jul 2;11(7):125. doi: 10.1038/s41408-021-00516-1 (PMC8253853; doi:10.1038/s41408-021-00516-1)
Supplement: Supplementary file 4 — Supplemental Tables [file 41408_2021_516_MOESM4_ESM.docx]

| **Supplementary Table 1.** Clinically significant pathogenic variants and variants of unknown significance from 3 pairs of samples | | | | | | |
| --- | --- | --- | --- | --- | --- | --- |
| ID | Chr:Pos | Ref/Alt | Gene Names | Sequence Ontology | HGVS c. (Clinically Relevant) | HGVS p. (Clinically Relevant) |
| A3465 | 12:46244652 | C/- | ARID2 | frameshift_variant | NM_152641.2:c.2746delC | NP_689854.2:p.Gln916Asnfs*11 |
| A3465 | 11:108235893 | G/T | ATM | stop_gained | NM_000051.3:c.8935G>T | NP_000042.3:p.Glu2979Ter |
| A3465 | X:39930247 | C/A | BCOR | stop_gained | NM_001123385.1:c.3217G>T | NP_001116857.1:p.Glu1073Ter |
| A3465 | 17:29677284 | G/T | NF1 | stop_gained | NM_001042492.2:c.7405G>T | NP_001035957.1:p.Glu2469Ter |
| A3465 | 19:10467379 | G/A | TYK2 | stop_gained | NM_003331.4:c.2482C>T | NP_003322.3:p.Gln828Ter |
| A8750 | 17:5051961 | G/T | USP6 | stop_gained | NM_004505.2:c.2542G>T | NP_004496.2:p.Gly848Ter |
| L2475 | 5:112178811 | TCCCACCTAATCTCAG/- | APC | frameshift_variant | NM_000038.5:c.7526_7541del | NP_000029.2:p.Pro2509Leufs*2 |
| L2475 | 1:27100331 | CCCCTTCTGGCAG/- | ARID1A | frameshift_variant | NM_006015.4:c.4050_4062delTGGCAGCCCCTTC | NP_006006.3:p.Gly1351Profs*126 |
| L2475 | 1:27101653 | TGTTGAAGCCACACAGCCTG/- | ARID1A | frameshift_variant | NM_006015.4:c.4944_4963del | NP_006006.3:p.Thr1649Glufs*42 |
| L2475 | 16:3777800 | GCTGGGGGTGTTCA/- | CREBBP | frameshift_variant | NM_004380.2:c.7235_7248delTGAACACCCCCAGC | NP_004371.2:p.Leu2412Glnfs*30 |
| L2475 | 16:3779193 | GGGGGCTGGGCCGGGGGTGG/- | CREBBP | frameshift_variant | NM_004380.2:c.5836_5855del | NP_004371.2:p.Pro1946Serfs*13 |
| L2475 | 22:41574670 | CAGTCCCAGCCCCCCCACTC/- | EP300 | frameshift_variant | NM_001429.3:c.6961_6980del | NP_001420.2:p.Gln2321Phefs*51 |
| L2475 | 4:55597498 | G/A | KIT | missense_variant | NM_000222.2:c.2146G>A | NP_000213.1:p.Asp716Asn |
| L2475 | 11:118375084 | CCTATAATACTGAGCT/- | KMT2A | frameshift_variant | NM_001197104.1:c.8480_8495del | NP_001184033.1:p.Tyr2827Ter |
| L2475 | 12:49426442 | CCAGGGCT/- | KMT2D | frameshift_variant | NM_003482.3:c.12039_12046delAGCCCTGG | NP_003473.3:p.Ala4014Serfs*23 |
| L3014 | 20:30956839 | C/A | ASXL1 | stop_gained | NM_015338.5:c.165C>A | NP_056153.2:p.Cys55Ter |
| L3014 | 3:105421044 | C/A | CBLB | missense_variant | NM_170662.3:c.1853G>T | NP_733762.2:p.Gly618Val |
| L3014 | 16:3843564 | C/T | CREBBP | missense_variant | NM_004380.2:c.1039G>A | NP_004371.2:p.Asp347Asn |
| L3014 | 22:41572351 | G/T | EP300 | missense_variant | NM_001429.3:c.4880G>T | NP_001420.2:p.Arg1627Leu |
| L3014 | 3:47059206 | G/T | SETD2 | stop_gained | NM_014159.6:c.7455C>A | NP_054878.5:p.Cys2485Ter |
| L3014 | 4:106197249 | G/T | TET2 | missense_variant | NM_001127208.2:c.5582G>T | NP_001120680.1:p.Gly1861Val |
| M7873 | 5:112177885 | GATTACTGGAAAAGTTC/- | APC | frameshift_variant | NM_000038.5:c.6597_6613del | NP_000029.2:p.Thr2200Ter |
| M7873 | 6:157099319 | -/A | ARID1B | frameshift_variant | NM_020732.3:c.255_256insA | NP_065783.3:p.His86Thrfs*146 |
| M7873 | 16:3900873 | G/A | CREBBP | stop_gained | NM_004380.2:c.223C>T | NP_004371.2:p.Arg75Ter |
| M7873 | 15:90631935 | G/A | IDH2 | missense_variant | NM_002168.2:c.418C>T | NP_002159.2:p.Arg140Trp |
| M7873 | 17:29560112 | G/- | NF1 | frameshift_variant | NM_001042492.2:c.3589delG | NP_001035957.1:p.Ala1197Glnfs*18 |
| M7873 | 9:36846904 | -/CCCGG | PAX5 | frameshift_variant | NM_016734.2:c.1035_1036insCCGGG | NP_057953.1:p.Tyr346Profs |
| M7873 | 12:112888237 | C/T | PTPN11 | missense_variant | NM_002834.3:c.253C>T | NP_002825.3:p.His85Tyr |
| M7873 | 17:7578380 | C/T | TP53 | missense_variant | NM_000546.5:c.550G>A | NP_000537.3:p.Asp184Asn |
| M7873 | 17:7578457 | C/T | TP53 | missense_variant | NM_000546.5:c.473G>A | NP_000537.3:p.Arg158His |
| M8807 | 7:151935871 | C/A | KMT2C | missense_variant | NM_170606.2:c.2573G>T | NP_733751.2:p.Trp858Leu |
| M8807 | 12:124829344 | C/T | NCOR2 | missense_variant | NM_006312.5:c.4513G>A | NP_006303.4:p.Glu1505Lys |

| **Supplementary Table 2.** Treatment | | | |
| --- | --- | --- | --- |
|  | **Late relapse**  **(< 10 years)** | **Very late relapse**  **(= or > 10 years)** | **All** |
| **Induction regimen of initial diagnosis**  COG/CCG regimen  SWOG 9400  BFM-based  CALGB | 14 (58)  3 (13)  5 (21)  2 (8) | 7 (58)  1 (8)  4 (33)  0 (0) | 21 (58)  4 (13)  9 (25)  2 (6) |
| **CR post initial induction** | 24 (100) | 12 (100) | 36 (100) |
| **Therapy at first relapse**  COG/CCC/R3  HyperCVAD  BFM  C10403  Others | 10 (42)  7 (29)  3 (12)  0 (0)  4 (17) | 3 (25)  6 (50)  0 (0)  2 (17)  1 (8) | 12 (33)  13 (36)  3 (8)  2 (6)  6 (17) |
| **CR post first salvage**  Yes  No | 23 (96)  1 (4) | 10 (83)  2 (17) | 33 (92)  3 (8) |
| **Allogeneic HCT post 1^st^ relapse**  Yes  No | 21 (87.5)  3 (12.5) | 7 (58)  5 (42) | 28 (78)  8 (22) |
| **Disease status at the time of HCT**  CR2  CR3  Relapse 2 | 14 (67)  6 (29)  1 (5) | 6 (86)  1 (14)  0 (0) | 20 (71)  7 (25)  1 (6) |
| **Conditioning regimen**  Myeloablative  Reduced Intensity/non-myeloablative | 19 (90)  2 (10) | 5 (71)  2 (29) | 24 (86)  4 (14) |
| **Donor**  Unrelated  Matched Sibling  Haplo-identical | 14 (67)  6 (29)  1 (5) | 5 (71)  2 (29)  0 (0) | 19 (68)  8 (29)  1 (4) |

| **Stem cells source**  Peripheral blood stem cells  Bone marrow | 17 (81)  4 (19) | 7 (100)  0 (0) | 24 (86)  4 (14) |
| --- | --- | --- | --- |

| **Novel therapies**  Blinatumomab  MRD  R/R  Inotuzumab  CAR T cell therapy  Venetoclax-navitoclax | 4 (17)  2 (8)  2 (8)  0 (0)  1 (4)  0 (0) | 5 (42)  1 (8)  4 (33)  2 (17)  2 (17)  2 (17) | 9 (25)  3 (8)  6 (17)  2 (6)  3 (8)  2 (6) |
| --- | --- | --- | --- |
